# Double anus in an *Ixodes scapularis* nymph, a medically important tick vector

**DOI:** 10.1186/s13071-021-04757-8

**Published:** 2021-05-11

**Authors:** Vikas Taank, Frank A. Lattanzio, Hameeda Sultana, Girish Neelakanta

**Affiliations:** 1 Department of Biological Sciences, Old Dominion University, Norfolk, VA USA; 2 Department of Physiological Sciences, Eastern Virginia Medical School, Norfolk, VA USA; 3 Center for Molecular Medicine, Old Dominion University, Norfolk, VA USA; 4 Present Address: Department of Biomedical and Diagnostic Sciences, College of Veterinary Medicine, University of Tennessee, Knoxville, TN 37996 USA

**Keywords:** Abnormalities, Anus, Microinjection, *Ixodes scapularis*

## Abstract

**Background:**

*Ixodes scapularis* ticks are medically important arthropod vectors that transmit several pathogens to humans. The observations of morphological abnormalities, including nanism, missing leg, extra leg, and gynandromorphism, have been reported in these ticks. In this study, we report the presence of two anuses in a laboratory-reared *I. scapularis* nymph.

**Results:**

Larval ticks were allowed to feed on mice and to molt to nymphs. Two anuses were observed in one of the freshly molted nymphs. Stereo and scanning electron microscopy confirmed the presence of two anuses in one nymph within a single anal groove.

**Conclusions:**

This report confirms the rare occurrence of double anus in *I. scapularis*.

**Graphic Abstract:**

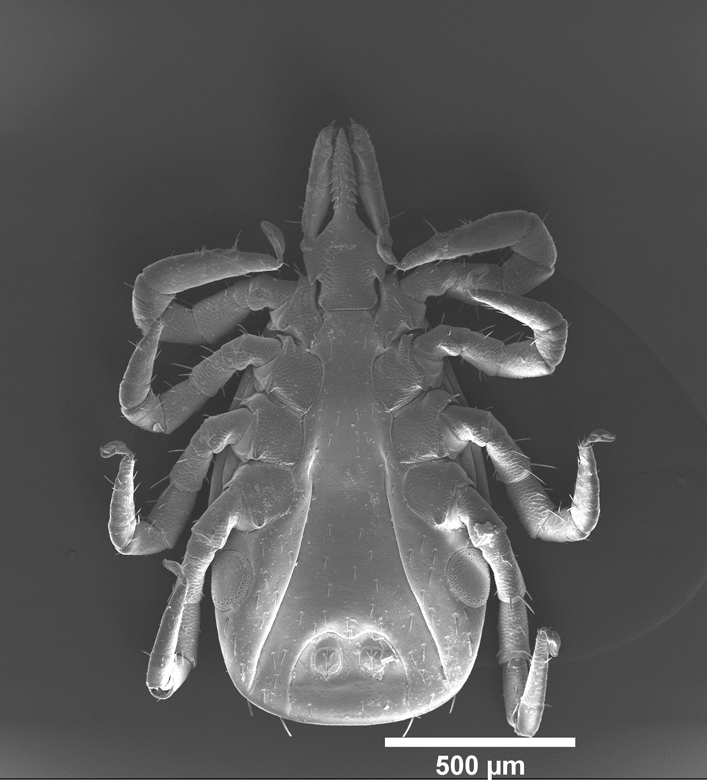

**Supplementary Information:**

The online version contains supplementary material available at 10.1186/s13071-021-04757-8.

## Background

Ticks such as *Ixodes scapularis* are medically important vectors that transmit several pathogens to humans [1, 2]. In nature, these ticks pass through several different developmental stages in their 2-year life cycle [3]. The six-legged larvae take a blood meal and molt into eight-legged nymphs. Nymphs then take a blood meal and molt into either adult male or female ticks. Female ticks take a blood meal, mate with male ticks, and lay eggs. Eggs then hatch into larvae and the life-cycle continues [3]. Ticks undergo several biotic and abiotic stress conditions during their life-cycle, with potential stress factors including, but are not limited to, changes in temperature, humidity, and lack of hosts to feed upon [3].

The first study that reported abnormalities in ticks was published in 1899 [4]. In the years that followed, several studies have reported morphological abnormalities in various hard and soft ticks including, but not limited to, species belonging to genera *Ixodes*,* Amblyomma*,* Hyalomma*,* Rhipicephalus*,* Dermacentor*,* Haemaphysalis*,* Ornithodoros*, and *Argas* [5–10]. The cause of abnormalities in ticks could be related to injuries in nature, somatic or germinal mutations, environmental factors, and exposure to insecticides and/or other chemicals during the developmental stages [5, 7–10]. It has also been reported that arthropod blood-feeding on exotic hosts could also result in abnormalities in ticks [11]. Abnormalities in ticks have been broadly classified into two categories: (i) local abnormalities, which include splitting of claws, tarsus, and femur, fusion of appendages, the absence of one or more legs, and defects in mouth parts; and (ii) general abnormalities, which include gigantism, gynandromorphism, nanism, double monsters, and asymmetry of the idiosoma [6].

The observations of local or general abnormalities in ticks are very rare, with the prevalence ranging from 0.028 to 0.2% in tick populations [12–14]. Kar et al. [12] reported that out of 18,667 ticks analyzed, only 33 specimens had abnormalities. In another study, Tovornik [14] reported that out of 53,930 ticks analyzed, only 15 specimens had abnormalities. In addition, Larson and Paskewitz [13] reported that out of 8800 nymphs analyzed, only three specimens had major abnormalities. *Ixodes scapularis* ticks are predominantly found in northeast and upper midwest parts of the USA [3]. Various studies have reported the presence of abnormalities such as nanism, two asymmetrical ticks (one with a missing leg and other with an extra leg), and gynandromorphism in field-collected *I. scapularis* ticks [13, 15, 16]. A study conducted by the New York State Department of Health and U.S. Department of Agriculture reported that out of more than 81,000 adult *I. scapularis* analyzed, only two specimens had abnormalities [16]. Collectively, the findings (from studies that analyzed samples of varying sizes) clearly suggest that abnormalities in ticks are very rare.

In this study, we report a local abnormality, namely, the presence of two anuses, in one of the laboratory-reared unfed nymphs. We noted that the two anuses were present within one anal groove. The observation of two anuses in *I. scapularis* provides further evidence of the presence of rare abnormalities in this group of ticks.

## Methods

### Tick colony maintenance and mice

Laboratory-reared *I. scapularis* ticks (larvae) obtained from a continuously maintained colony at BEI Resources (Manassas, VA, USA))/U.S. Center for Disease Control and Prevention (CDC) were used in this study (CDC catalog no. NR-44115 for larvae of live wild-type *I. scapularis*). *Ixodes scapularis* ticks were flagged from vegetation in 2003 in Rhode Island, USA. C3H/HeN mice (female, 4–6 weeks old; Charles River Laboratories, Inc., Wilmington, MA, USA) were used in this study. Larvae were allowed to feed on naïve mice and allowed to molt into nymphs, generating uninfected nymphs. Tick rearing was conducted in an incubator at 23 ± 2 °C and 90–95% relative humidity under a 14/10-h light/dark photoperiod regimen, as described in our previous studies [17–19].

### Bacterial isolate, *A. phagocytophilum* host cell-free dense core isolation, and tick microinjection

*Anaplasma phagocytophilum* isolate NCH-1 (obtained from BEI Resources) was used in this study. A host cell-free dense core form of *A. phagocytophilum* (*Ap*-DC) was isolated from confluent HL-60 cells with > 70% infection by sonication, followed by multiple differential centrifugation steps as described in previous studies [19, 20]. To analyze if physical disruption (*via* microinjection) of the anal pore affects the viability of a tick with double anus, *Ap-*DC was introduced by microinjection into the anal pore, as described in our recent study [19]. Microinjected ticks were observed at 2 h post-microinjection under a stereomicroscope and pictures were taken. Thereafter, the tick with double anus and a normal tick with a single anus were stored in fresh 70% ethanol in 1.5-ml Eppendorf tube at 4 °C until other microscopy studies were conducted.

### Stereomicroscopic images and video

To assess the overall health of the live ticks after microinjection, we performed stereomicroscopic visualization. Live ticks were immobilized by placing the ticks (on their dorsal side) on a microscope slide on a double-sided tape. Video and Images were captured using a Nikon SMZ74 5 T stereomicroscope (Nikon, Tokyo, Japan) equipped with AmScope CMOS camera attachment (AmScope, Irvine, CA USA) and ToupView Image Capture software 3.7 (AmScope). Image J software (U.S. National Institutes of Health, Bethesda, MD, USA) was used for image enhancements.

### Scanning electron microscopy

To analyze the structural features of the tick with double anus in detail, we studied the tick samples by scanning electron microscopy (SEM). Sample preparation and SEM microscopy were performed at the Virginia Commonwealth University Microscopy Facility (Richmond, VA, USA). Briefly, tick samples were removed from storage in 70% ethanol and dehydrated through a series of ethanol solutions (70, 80, 95, and 3 changes of 100%), following which they were critical point dried using an Autosamdri-814 Critical Point dryer (Tousimis Research Corp., Rockville, MD, USA). The samples were then mounted on standard aluminum SEM mounts and sputter-coated with gold–palladium, using an EMS 550 × sputter coater (Electron Microscopy Sciences, Hatfield, PA, USA). Electron micrographs were captured using a Zeiss EVO 50 XVP scanning electron microscope (Carl Zeiss Microscopy GmbH, Jena, Germany) operating at 10.00 kV.

## Results and discussion

**Fig. 1:**
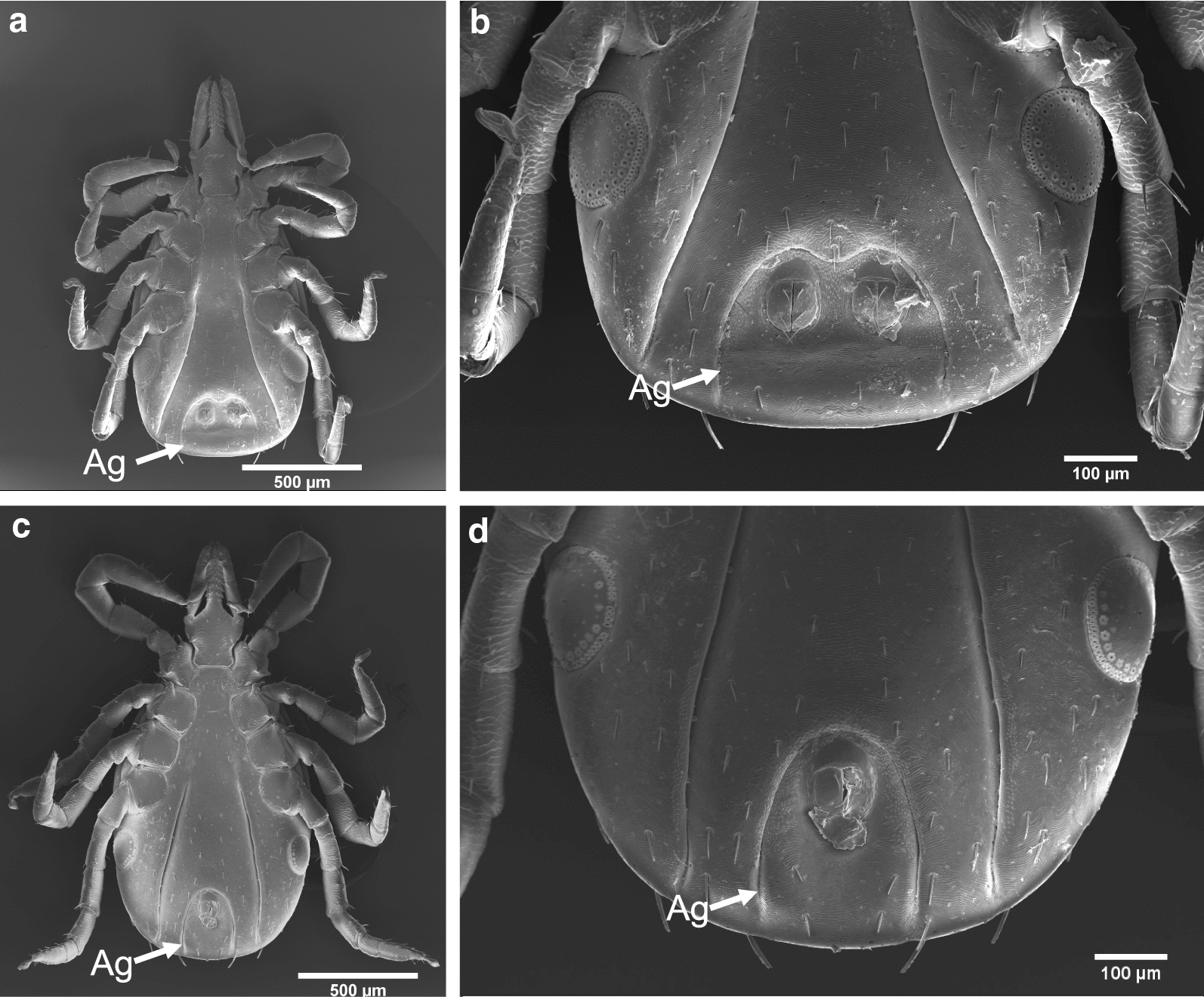
Scanning electron micrographs of the *Ixodes scapularis* nymph with two anuses and a normal nymph with a single anus.** a**,** b** Abnormal tick. Ventral side of whole tick (**a**) and enlarged portion of the same tick (**b**) with two anuses.** c**,** d** Normal tick. Ventral side of whole tick (**c**) and enlarged portion of the same tick (**d**) with one anus. Scale bar:** a**,** c** 500 µm,** b**,** d** 100 µm.* Ag* Anal groove

Larvae obtained from BEI resources were allowed to feed on naïve C3H/HeN mice and the fed larvae were allowed to molt into nymphs. We noted that one of the freshly molted nymphal ticks contained two anuses. SEM images clearly revealed the presence of two anuses in the abnormal nymph (Fig. 1a, b) and only a single anus in the normal nymph (Fig. 1c, d). In addition, the double anuses were found to be in a single anal groove. The abnormal nymph presented a more extensive anal groove with the presence of two anal pores of similar size (Fig. 1a, b). The diameter of both anal pores in the abnormal nymph was between 65 and 67 µm. The two anuses were separated by approximately 115 µm, and the largest width of the anal groove was measured to be approximately 315 µm. In comparision, the single anal pore in the normal nymph tick was 75–77 µm in diameter and the anal grove was apporximately 210 µm wide.

In a recent study, we developed a novel method for the generation of *Anaplasma phagocytophilum*-infected ticks via microinjection [19]. Microinjection is a routine technique to introduce pathogens, double-stranded RNA, inhibitors, or metabolites into ticks [18, 19]. Two types of microinjections can be performed, one into the anal pore and the other into the body [18, 19]. Microinjections will result in the physical disruption of the body or anus in ticks. However, when properly done, ticks can sustain and survive these two types of microinjections. As we noted two anuses in a nymphal tick, we questioned whether physical disruption of the anus in the tick with the double anuses would have any effect on tick viability. To determine if the tick with double anus can sustain microinjection, we injected *A. phagocytophilum*-dense core form (*Ap*-DC) into one of the anuses. A 2 h post-microinjection, ticks were visualized under a stereomicroscope to assess the overall health and fitness. The tick with two anuses appeared to be healthy and mobile (Additional file 1: Video S1).

We have published several studies using *I. scapularis* ticks obtained from several sources including BEI resources (CDC), the Oklahoma Tick Facility, and the Connecticut Agricultural Experimental Station and Yale School of Public Health (Yale University) [17, 18, 21–26]. Until this finding, we had not observed any tick containing double anus, suggesting a rare abnormality in this group of ticks. To our knowledge, in this study we did not find any other local abnormalities in the double anus-containing tick.

In a study on *Amblyomma variegatum*, Latif et al. [27] identified abnormalities in 16 out of 5000 larvae. These authors observed external abnormalities, such as partial twinning of the posterior region of the body with the presence of two anal pores and two genital grooves and also identified the presence of two anal pores in nymphal and adult stages of these ticks. Of the 16 abnormal ticks identified in the study [27], 11 engorged successfully on rabbits, suggesting that abnormalities such as presence of two anal pores may not affect tick feeding. The tick with two anuses identified in our study was noted to be viable and mobile even after 2 h post-microinjection, suggesting that the presence of this local abnormality may not affect mobility of these ticks. We hypothesize that the two anuses in an unfed nymphal tick identified in our study is presumably related to somatic or germinal mutations that could have occurred during the development from the egg to thelarval stage of this tick. This abnormality might have retained during molting from larval to nymphal stage.

Larson and Paskewitz [13] reported four abnormalities in field-collected *I. scapularis* ticks, including schizomely of the third leg in the first tick, slight asymmetry and distorted point on the alloscutum in the second tick, ectromely of the third leg on the right side of the third tick, and extreme asymmetry and enlarged coxae in the fourth tick. Prusinski et al. [16] reported gynandromorphism for the first time in *I. scapularis*. Soghigian et al. [15] reported the first evidence of dwarfism in *I. scapularis* that was found parasitizing a human host. Our report on the presence of double anus in one unfed nymphal tick further adds to the list of abnormalities observed in *I. scapularis*. *Ixodes scapularis* is a medically important vector for several human pathogens [1, 2]. The findings from the current study and other published studies that reported abnormalities in *I. scapularis* [13, 15, 16] provide evidence for further studies to focus on looking at abnormalities in all field-collected ticks.

## Conclusions

This report confirms the rare occurrence of double anus in a laboratory-reared medically important *I. scapularis* tick. Future studies that focus on both pathogen surveillance and looking at abnormalities in field-collected ticks may provide information on whether abnormalities in ticks have any implications in disease transmission and vector competency.

## Supplementary Information

**Additional file 1**: **Video S1**. Live stereomicroscope ventral view of the abnormal Ixodes scapularis nymph with two anuses (right side) and a normal tick (left side) with a single anus. The scale bar on the right side indicates 1 mm.

## Data Availability

All data that support the findings reported in this study are included in the main article file and in the supplementary information file.

## Abbreviations

DC: Dense core form of *Anaplasma phagocytophilum*
HL-60: Human leukemia cell line
SEM: Scanning electron microscopy
